# Antiangiogenic Activity and Pharmacogenomics of Medicinal Plants from Traditional Korean Medicine

**DOI:** 10.1155/2013/131306

**Published:** 2013-07-22

**Authors:** Ean-Jeong Seo, Victor Kuete, Onat Kadioglu, Benjamin Krusche, Sven Schröder, Henry Johannes Greten, Joachim Arend, Ik-Soo Lee, Thomas Efferth

**Affiliations:** ^1^Department of Pharmaceutical Biology, Institute of Pharmacy and Biochemistry, Johannes Gutenberg University, Staudinger Weg 5, 55128 Mainz, Germany; ^2^Department of Biochemistry, Faculty of Science, University of Dschang, Cameroon; ^3^HanseMerkur Center for Traditional Chinese Medicine, University Hospital Eppendorf, Hamburg, Germany; ^4^Biomedical Sciences Institute Abel Salazar, University of Porto, Portugal; ^5^Heidelberg School of Chinese Medicine, Heidelberg, Germany; ^6^College of Pharmacy, Chonnam National University, Gwangju, Republic of Korea

## Abstract

*Aim*. In the present study, we investigated the antiangiogenic properties of 59 plants used in traditional Korean medicine. Selected phytochemicals were investigated in more detail for their modes of action. *Methods*. A modified chicken-chorioallantoic-membrane (CAM) assay using quail eggs was applied to test for antiangiogenic effects of plant extracts. A molecular docking *in silico* approached the binding of plant constituents to the vascular endothelial growth factor receptors 1 and 2 (VEGFR1, VEGFR2). Microarray-based mRNA expression profiling was employed to correlate the 50% inhibition concentrations (IC_50_) of a panel of 60 NCI cell lines to these phytochemicals. *Results*. Extracts from *Acer mono* leaves, *Reynoutria sachalniensis* fruits, *Cinnamomum japonicum* stems, *Eurya japonica* leaves, *Adenophora racemosa* whole plant, *Caryopteris incana* leaves-stems, and *Schisandra chinensis* stems inhibited angiogenesis more than 50% in quail eggs. Selected phytochemicals from Korean plants were analyzed in more detail using microarray-based mRNA expression profiles and molecular docking to VEGFR1 and VEGFR2. These results indicate multifactorial modes of action of these natural products. *Conclusion*. The antiangiogenic activity of plants used in traditional Korean medicine implicates their possible application for diseases where inhibition of blood vessel formation is desired, for example, cancer, macular degeneration, diabetic retinopathy and others.

## 1. Introduction

Traditional medicinal plants belong to the characteristics of most cultures on this earth. Medicinal plants helped to secure survival of our ancestors in a noncivilized world, which was not dominated by the technological achievements of the 20th and 21st centuries. Medicinal plants were not only indispensable for as basic health care of indigenous tribes in former ages as modern medicine was not in place. Even nowadays, phytotherapy is still used by a majority of the world's population. Over 50,000 plants would possess therapeutic virtues in the world and about 80% of human use herbal medicines at least once in their life [1, 2]. The pharmacological screening of plants is an important mean for the discovery of new, safe, and effective drugs in classical pharmacology [3]. Hence, it comes as no surprise that research on medicinal plants and natural products derived from them experiences a thriving revival in the past years. 

Complementary and alternative medicine and traditional medicines are well acknowledged among the general population in industrialized countries [4, 5]. Many patients use complementary and alternative medicine, frequently without the knowledge of their doctors. Therefore, there is an urgent need for quality-controlled and safe but also effective products from complementary and alternative medicine. Clinical trials and cellular and molecular mechanistic studies on medicinal herbs will help to improve their rational use and to better understand their modes of action. This was the motivation for many scientists from pharmacy and pharmacology, including our own group, to investigate the bioactivity of medicinal plants and phytochemicals isolated from them using techniques of molecular pharmacology and molecular biology [6–11]. With a strong commitment to traditional Chinese medicine [12–14] and traditional African medicine in the past years [15–17], we now focus on traditional Korean medicine.

Traditional Korean medicine is widely used in Korea and is the primary health care system for more than 20% of the population [18, 19]. Demographic studies confirm that traditional Korean medicine flourishes in modern Korea. One regional survey found that 36% of the Korean population had used complementary and alternative medicine in a 5-year period, although traditional Korean medicine use was not specified [20]. Forty percent of hypertensive patients used complementary and alternative medicine including traditional Korean medicine after discharge from hospitals [18]. A South Korean national survey was performed among 79% of people who are older than 18 years old and have health problems within one year. The result showed that 40% of those did not do anything, 23% of them visited western doctors' offices or western hospitals, and 19% used complementary and alternative medicine only (including traditional Korean medicine), with 18% using both western medical and TKM service [20].

It was reported that excessive angiogenesis is an important factor of the pathogenesis of many industrialized western countries [21]. Plants with anti-angiogenesis properties are therefore of considerable importance for diseases such as cancer, macular degeneration, diabetic retinopathy, and others [22–25]. 

Based on the antiangiogenic activity of the plant extracts in a modified *in vivo* chicken chorioallantoic membrane assay, selected phytochemicals were analyzed in more detail. A molecular docking approach was applied to investigate *in silico* the binding of selected phytochemicals to the vascular endothelial growth factor receptor (VEGFR1, FLT1) as an important angiogenic factor.

As previously shown for other antiangiogenic drugs [26], the microarray-based mRNA expression of VEGFR1/FLT1 and 89 other angiogenesis-regulating genes was correlated to the IC_50_ values of 60 cell lines of the National Cancer Institute, USA, for selected phytochemicals derived from traditional Korean medicine to identify possible cellular factors associated with their antiangiogenic activity. 

## 2. Materials and Methods

### 2.1. Plant Material and Extraction

Medicinal plants used in the present work were collected at different localities of South Korea and provided by Professor Ik-Soo Lee (College of Pharmacy, Chonnam National University, Gwangju, South Korea). The plants were identified at the national herbarium, where voucher specimens were deposited under the references numbers (see Supplementary Table 1 in Supplementry Materials available online at http://dx.doi.org/10.1155/2013/131306). The extraction of the air-dried and powdered plant material was conducted using methanol (HPLC grade) with either ASE 300 (Dionex) or a sonicator (Branson Ultrasonics) at 50°C. The extracts were then conserved at 4°C until further use.

### 2.2. Angiogenesis Test

#### 2.2.1. Cultivation of Quail Eggs

Quail eggs were cultured according to a described method [27]. Briefly, fertilized quail eggs were incubated for 70 h at 38°C and 80% relative humidity. After 70 h of incubation the eggs were opened. For this purpose, the eggs were placed in a vertical position to guarantee that the embryo floats in the upper part of the egg. Afterwards, a hole was cut into the top of the egg and the complete content of the egg, was transferred into a Petri dish. By using this method, it could be guaranteed that the albumin gets first into the Petri dish followed by the yolk with the embryo on top without exposing the embryo to shock forces which could damage the vitelline membrane.

#### 2.2.2. Chicken Chorioallantoic Membrane Assay (CAM Assay)

Plant extracts were tested for their antiangiogenic effects on quail eggs as previously described for chicken eggs with modifications [28, 29]. Briefly, the explanted embryo was placed in an incubator for 2 h at 38°C to acclimatize it to the new ambience. Subsequently, the test substances were placed on the CAM. Therefore, 2% agarose solution was prepared and mixed 1 : 10 with the plant extract prior diluted in DMSO 0.1% final concentration. The final concentration of the extract was 10 *μ*g/mL. Pellets with 0.1% DMSO served as control. The agarose-pellets were then placed on the CAM after they cooled down to room temperature. The Petri dishes with the quail embryos were placed in the incubator again and incubated at 38°C and 80% relative humidity for 24 h before documenting the effect of the applied substances.

Imaging of the vascularized quail eggs was performed using a digital camera with 3x magnification objective (Canon eos 500 with a Canon mp-e 65 2.8 macro objective). For illumination, a mercury arc lamp was used which provided a high fraction of blue and UV light to obtain good contrast values between yolk and vessels. The pictured image section had a size of 5 × 5 mm. Following image acquisition, quantitative analysis was performed using a routine software which was written in the ImageJ-macro language, then the total small vessel number (or area) was then determined by the system, and the percentage inhibition of vascularization was calculated [30]. 

### 2.3. Correlation of Angiogenesis-Regulating Gene Expression with Cytotoxicity of Tumor Cell Lines

The mRNA microarray hybridization of the NCI cell line panel has been described [31, 32], and the data has been deposited at the NCI website (http://dtp.nci.nih.gov/). The performance of the COMPARE and hierarchical cluster analyses using mRNA-based microarray data of the database of the National Cancer Institute, USA, has been previously described by us [33]. A set of 89 genes were chosen because of their involvement in angiogenic processes [34]. The microarray data of this set of genes was exemplarily validated by real-time RT-PCR [26]. 

### 2.4. Statistics

Pearson's correlation test was used to calculate significance values and rank correlation coefficients as relative measure for the linear dependency of two variables. This test was implemented into the WinSTAT Program (Kalmia). The one-way ANOVA at 95% confidence level was used for statistical analysis.

### 2.5. Molecular Docking

Human vascular endothelial growth factor receptor 1 tyrosine kinase domain (VEGFR1-TK) structure was retrieved from PDB database (PDB code: 3HNG), which was submitted in complex with N-(4-chlorophenyl)-2-[(pyridin-4-ylmethyl)amino]benzamide). ChemSpider and PubChem were referred for the 3D structures of control drugs and the Korean medicine compounds. Molecular docking calculations were performed with AutoDock4 [35]. Axitinib, which is an antiangiogenic compound and a known VEGFR1 and VEGFR2 inhibitor, was selected as the control drug to compare the binding energies and the docking sites of the candidate ligands. The residues of VEGFR1, which the N-(4-chlorophenyl)-2-[(pyridin-4-ylmethyl)amino]benzamide and control drugs in the literature make hydrogen bond with, were selected for the defined docking. Drug binding residues of VEGFR1 were identified as Val841, Ala859, Lys861, Glu878, Leu882, Val892, Val909, Cys912, Leu1029, and Asp1040. Furthermore, VEGFR2 PDB structure (PDB code: 3U6J), which was submitted in complex with a pyrazolone inhibitor (N-{4-[(6,7-dimethoxyquinolin-4-yl)oxy]-3-fluorophenyl}-1,5-dimethyl-3-oxo-2-phenyl-2,3-dihydro-1H-pyrazole-4-carboxamide), was used. The drug interaction residues for VEGFR2 were Leu 840, Ala 866, Lys 868, Leu 889, Ile 892, Phe 918, Cys 919, Leu 1019, Leu 1035, Cys 1045, Asp 1046, and Phe 1047.

Grid maps were created covering those residues. For the docking calculations, the number of energy evaluations was set to 2,500,000 and the number of runs was set to 100. The lamarckian Genetic Algorithm was chosen for the docking calculations. For the visualization of the docking results, AutoDock Tools and Visual Molecular Dynamics were used. The surface representation image showing the binding pocket of human VEGFR1-TK was made with VMD software developed with NIH support by the theoretical and computational biophysics group at the Beckman Institute, University of Illinois at Urbana-Champaign.

## 3. Results

### 3.1. Antiangiogenic Activity *In Vivo*

**Figure 1:**
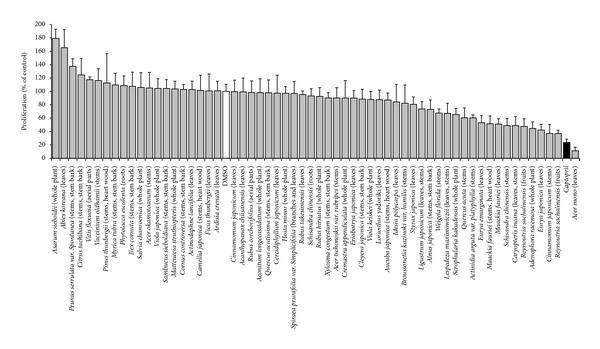
Antiangiogenic effects of the 59 Korean plant extracts (10 *μ*g/mL) on the growth of blood capillaries on the CAM of quail eggs. Mean values ± SD of each five eggs are shown. DMSO: solvent control (white bar); captopril: positive control (10 *μ*g/mL; black bar).

Out of 59 plant extracts tested in the CAM assay, seven samples showed significant inhibition (>50%) of angiogenesis. They include extracts from *Acer mono *leaves, *Reynoutria sachalniensis *fruits, *Cinnamomum japonicum *stems, *Eurya japonica *leaves, *Adenophora racemosa* whole plant, *Caryopteris incana *leaves-stems, and *Schisandra chinensis* stems (Figure 1). 

**Figure 2:**
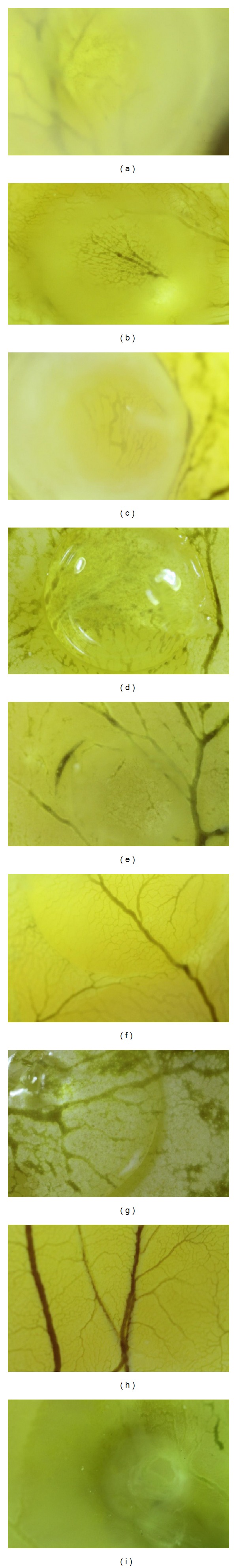
Representative images of the effect of antiangiogenic Korean plant extracts (10 *μ*g/mL) on the growth of blood capillaries on the CAM of quail eggs. The tested extracts were from: *Acer mono* leaves (a); *Reynoutria sachalniensis* fruits (b); *Cinnamomum japonicum* stems (c); *Eurya japonica* leaves (d); *Adenophora racemosa* whole plant (e); *Caryopteris incana *leaves stems (f); *Schisandra chinensis* stems (g); DMSO or solvent control (h); captopril as positive control (i).

Representative images of the effect of antiangiogenic Korean plant extracts (10 *μ*g/mL) on the growth of blood capillaries on the CAMs of quail eggs are shown in Figure 2. 

### 3.2. Correlation of mRNA Expression of Angiogenic Genes with IC_**50**_ Values of NCI Cell Lines for Phytochemicals from Korean Plants

**Figure 3:**
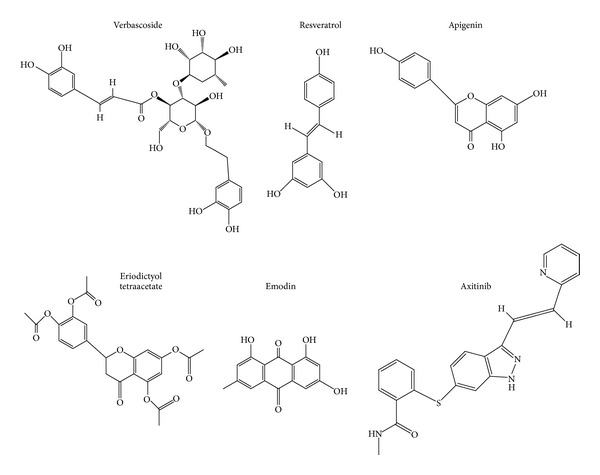
Chemicals structures of selected phytochemicals derived from Korean medicinal plants.

**Figure 4:**
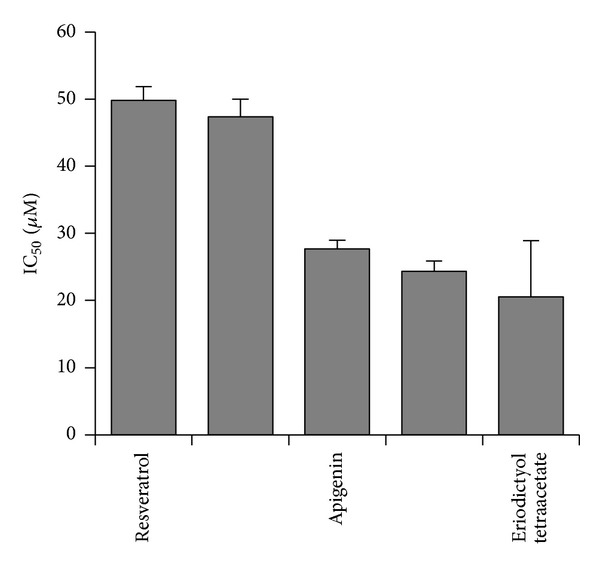
Cytotoxic activity of selected phytochemicals derived from Korean medicinal plants for tumor cell lines from the NCI cell line panel.

As a next step, we searched the literature on chemical constituents of antiangiogenic Korean plants (Table 1). Then, we mined the NCI database for these compounds (http://dtp.nci.nih.gov/). Five compounds were found in the database and were exemplarily selected as possible antiangiogenic candidate compounds, that is, verbascoside, apigenin, emodin, resveratrol, and eriodictyol tetraacetate (Figure 3). Axitinib is a known VEGFR inhibitor and served as control drug. The average IC_50_ values over the entire range of NCI cell lines are shown in Figure 4.

The IC_50_ values of these phytochemicals were correlated with the baseline mRNA expression levels of 89 genes involved in angiogenic pathways for the NCI panel of tumor cell lines by the Pearson rank correlation test. Only those genes, whose expression correlated with *R* > 0.3 or *R* < −0.3 with the IC_50_ values of the five compounds were considered for further analyses (Table 2).

**Figure 5:**
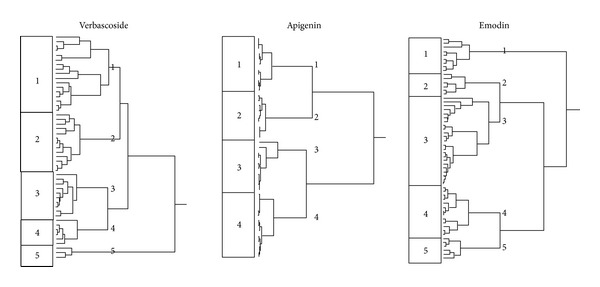
Dendrograms obtained by hierarchical cluster analysis of microarray-based expressions of angiogenesis-regulating genes for selected phytochemicals derived from Korean medicinal plants for tumor cell lines from the NCI cell line panel. The dendrograms were obtained by clustering using the WARD method. Extended versions of these dendrograms showing the exact positions of each cell line are included as Supplementary Material.

The gene expressions of these genes were then subjected to hierarchical cluster analysis. The dendrograms for verbascoside, apigenin, and emodin are shown in Figure 5. To investigate whether these gene expression profiles contain relevant information, we correlated them with the distribution of IC_50_ values for these three compounds of the cell lines. The IC_50_ values themselves were not used for generation of the cluster dendrograms. Therefore, we could address the question whether or not the gene expressions alone predicted the response of the cell line panel to these phytochemicals. As shown in Table 3, the distribution of the cell lines sensitive or resistant to the three natural products was significantly different, indicating that these angiogenesis-regulating genes indeed determined the response of tumor cells to verbascoside, apigenin, and emodin. These analyses were also performed for resveratrol and eriodictyol tetraacetate, but significant relationships were not found, indicating that these gene expression profiles were not predictive for response of tumor cell lines to these two compounds (Table 3).

### 3.3. Molecular Docking of Phytochemicals from Korean Plants to VEGFR1 and VEGFR2

Since antiangiogenic effects may not only be mediated by up- or downregulation of gene expressions but also by direct binding to angiogenic target molecules, we addressed the question whether the five selected phytochemicals may bind to VEGFR1 and VEGFR2. For this reason, we applied an *in silico* molecular docking approach. As a control drug, we used axitinib, a synthetic small molecule inhibitor which binds to defined pharmacophores of VEGFR1 and VEGFR2. Remarkably, all five natural products bound to the same pharmacophores as axitinib, albeit at lower binding affinities (Table 4). Eriodictyol tetraacetate showed the lowest binding energy for both VEGFR1 and VEGFR2. Quercetin might be an efficient VEGFR1 inhibitor since it made hydrogen bond with drug binding residues (Glu878 and Cys912) with low binding energy. Apigenin was observed to make hydrogen bond with drug binding residues on VEGFR1 (Glu878, Cys912, and Asp1040) and VEGFR2 (Lys868, Cys919, Asp1046) like axitinib. Thus, apigenin seems to be a promising candidate as an antiangiogenic compound. Moreover, it was found that resveratrol interacted with VEGFR1 and VEGFR2 with high affinity and made hydrogen bond with drug binding residues (Glu878 and Cys912 on VEGFR1, Lys868, Cys919, and Asp1046 on VEGFR2). Besides, verbascoside bound to VEGFR2 (−9.99 kcal/mol) with higher affinity than VEGFR1 (6.93 kcal/mol). Emodin showed moderately low binding energies compared to other compounds.

**Figure 6:**
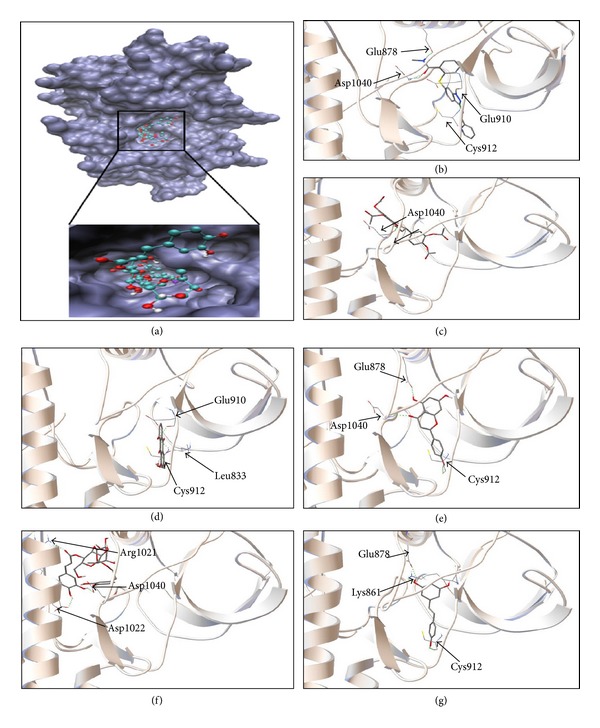
Docking studies of candidate antiangiogenic compounds. (a) Docking of 6 compounds into the binding site of VEGFR1-TK (PDB code: 3HNG in blue surface representation). The compounds occupy the same binding site as axitinib, a known antiangiogenic compound (in violet). Docked structure of axitinib (b), eriodictyol tetraacetate (c), emodin (d), apigenin (e), verbascoside (f), and resveratrol (g) in VEGFR1-TK binding pocket. The residues involved in hydrogen bond interaction are labeled, and hydrogen bonds are shown as green dots. Axitinib is a known VEGFR-TK inhibitor and was used as control drug.

The binding of the five phytochemicals and axitinib to VEGFR1 is shown in Figure 6. Similar binding modes were found for VEGFR2 (data not shown). 

## 4. Discussion

### 4.1. Antiangiogenic Activity *In Vivo*

Antiangiogenic compounds are gaining more and more interest as a new approach in the prevention and treatment of cancer and inflammatory diseases [48]. The CAM assay is a sensitive, easily feasible, and cheap *in vivo* test for investigations of the antiangiogenic potential of individual compounds and plant extracts [49]. The assay does not only provide information on the efficacy of test samples *in vivo* but also on their toxicity *in vivo*. 

To the best of our knowledge, their antiangiogenic property is being reported here for the first time. The best antiangiogenic effect was recorded with the extract from *Acer mono *(11.14% proliferation), this activity being better than that of captopril (23.54% proliferation), highlighting its possible importance in cancer therapy. Captopril served as control drug, since its antiangiogenic activity is well known and the drug also inhibited angiogenesis in the CAM-assay [50, 51]. *Schisandra chinensis* exhibited a good but different extent of angiogenesis inhibition with both leaves and stems extracts, strengthening the hypothesis that it is necessary to screen various plant organs when evaluating their pharmacological activities. A comparison of our Korean plant extracts showed that there was no correlation between cytotoxicity and antiangiogenic activity [52]. Therefore, these extracts might not only be used to inhibit angiogenesis in tumors but also for treatment of noncancerous diseases such as diabetic retinopathy or macular degeneration. It has been shown during the past years that therapeutic antibodies which target VEGF are not only active in cancer but are also a considerable potential for ophthalmologic applications [23]. It is reasonable to speculate that plant extracts with antiangiogenic properties may not only be candidate for cancer therapy but also for therapeutic applications in ophthalmology. 

### 4.2. Microarray-Based Gene Expression Profiling

In the present investigation, the IC_50_ profiles of five phytochemicals for the panel of 60 cell lines of the National Cancer Institute (NCI), USA, were correlated with the microarray-based expression profiles of the cell lines. The intention was to identify molecular determinants which predict sensitivity or resistance of tumor cells to these compounds. This concept was developed in the 1990s at the Developmental Therapeutics Program of the NCI to extract meaningful information of large-scale drug screenings [53]. During the past years, this concept provided a fertile ground to unravel mechanisms of action of new drugs and to use gene expression profiles for the prediction of chemosensitivity of tumor cells [54–56]. We applied this approach to gain insight of determinants of activity of natural products derived from traditional Chinese medicine, for example, homoharringtonine, artemisinin, cantharidin, arsenic trioxide, and others [36–40, 57]. 

In the present investigation, we focused on compounds derived from traditional Korean medicine. It was a striking feature that genes with diverse functions correlated with the response of the NCI cell lines to phytochemicals (verbascoside, apigenin, emodin, quercetin, eriodictyol, and resveratrol). This result may be taken as a hint that these natural products affect several targets and intracellular signaling pathways. This hypothesis is supported by similar observations of other authors. 

Emodin inhibits tumor growth *in vitro* and *in vivo* [41, 42]. Several proteins involved in angiogenesis have been associated with this effect, including matrix metalloproteinases 2 and 9, basic fibroblast growth factor, urokinase plasminogen activator, plasminogen inhibitor 1, the extracellular signal-regulated kinases 1 and 2 (ERK1/2), and the chemokine CXCR4 receptor [41, 42, 58, 59]. Furthermore, emodin inhibits the phosphorylation of the VEGF receptors 1, 2, and 3 [60]. 

Apigenin inhibits angiogenesis by inhibiting VEGF and HIF-1 expression via the PI3 K/AKT/P70S6 K1 and HDM2/p53 pathways [61, 62]. Further antiangiogenic mechanisms are downregulation of type I collagen, vimentin, matrix metalloproteinase 8, and of the cytokine IL6/STAT3 pathway [63, 64]. 

Angiogenesis is inhibited by resveratrol both *in vitro* and *in vivo* [65–67]. Several mechanisms have been unraveled, for example, downregulation and/or inhibition of VEGF, HIF-1*α*, Flk-1, and Src [68, 69]. Various signaling pathways contribute to inhibition of angiogenesis such as the eukaryotic elongation factor-2 kinase-regulated pathway, the GSK3*β*/*β*-catenin/TCF-dependent pathway, NF-*κ*B-related signaling, and cytokine signaling (IL8/CXCL8) [43, 44, 70, 71]. The antiangiogenic activity of verbascoside and eriodictyol tetraacetate has not been described yet.

Microarray analyses have been previously performed for emodin, verbascoside, and resveratrol and a huge number of genes have been found to be regulated by treatment with these compounds [45–47, 72–74]. Together with the microarray data of the present investigation, these results further emphasize the multifactorial activity of natural products. While some scientists from conventional academic medicine have called natural products as “dirty drugs” for their multiple modes of action, the past years of intense research on synthetic and monospecific drugs showed that synthetic drugs are not superior. Tumor cells readily develop resistance to monospecific drugs, for example, by point mutations in the corresponding target proteins preventing drug binding, by downregulation of target gene expression, or by activation of alternative signaling routes and bypassing of inhibited pathways [75]. The probability is much less that tumor cells escape treatment with multifactorial drugs, since resistance to one mode of drug action does not affect the drug's activity on other cellular signaling pathways. The fact that organisms developed rather multi- than monotarget compounds during evolution of life on earth may be taken as a clue that the concept of multitargeted therapy is superior [76, 77].

### 4.3. Molecular Docking of VEGFR1 and VEGFR2

Antiangiogenic compounds may not only exert their blood vessel inhibiting effects by up- or downregulation of angiogenesis-regulating genes, but also by targeting and binding to key regulators of angiogenesis. The specific targeting of growth factor receptors by therapeutic antibodies and small molecules is currently one of the most thriving fields in drug development with a plethora of new drugs on the market. This is also true for antiangiogenic therapies [22]. VEGFRs are exquisite targets to inhibit angiogenesis. Inhibition of the tyrosine kinase activity of VEGFR by small molecules leads to blockage of VEGFR-related downstream-signaling pathways, hence, inhibition of blood vessel sprouting. 

The number of VEGFR inhibitors was steadily increasing over the past few years [78]. One of them is axitinib, which specifically binds to all three VEGF receptors, VEGFR1, VEGFR2, and VEGFR3 [79, 80]. Therefore, this drug served as control for our bioinformatical docking studies. The idea was to investigate whether or not the five selected phytochemicals from Korean plants might bind to the same pharmacophore as axitinib. As the crystal structure of VEGFR3 was not available in the PDB database, we only analyzed VEGFR1 and VEGFR2. As expected, axitinib was predicted to bind with high affinity to both receptors, which is indicated by low free binding energies (<−12 kcal/mol). Molecular docking of verbascoside, apigenin, emodin, quercetin, eriodictyol tetraacetate, and resveratrol yielded free binding energies in a range from −6 to −9 kcal/mol. This indicates that these phytochemicals may bind to the receptors at lower affinity than axitinib. It can be speculated that these compounds efficiently inhibit angiogenesis by combining different mechanisms, such as VEGFR binding as well as up/downregulation of angiogenic genes and proteins. Therefore, these natural products may be efficient angiogenesis inhibitors, even if they bind with lower affinities to VEGF receptors than axitinib. For drug development, these phytochemicals may serve as lead compounds to synthesize novel derivatives with improved binding properties to VEGF receptors. 

## 5. Conclusion

We estimate medicinal plants in general and especially plants from traditional Korean medicine as valuable and indispensable resources for the development of new drugs and the rational use of phytotherapy. This point of view is supported by a comprehensive survey of the NCI, USA, showing that the vast majority of clinically established cancer drugs during the past three decades were based on natural products [81]. It can be expected that natural products and evidence-based complementary and alternative therapies such as inhibition of angiogenesis by Korean medicinal plants will lead to considerably improve the treatment of patients in the future.

## Supplementary Material

EST sequencing and electronic databases are used for gene profiling by F. V. Peale Jr. (Peale and Gerritsen 2001). Briefly, EST sequencing is creating cDNA libraries from tissues or cells of interest, randomly select clones, and perform a single sequencing reaction on each clone (Adams et al. 1991). The EST approach has been used to identify what can be the most expressed sequences in the human genome. This information can be applied in many ways, for instance, the tissues from the EST libraries are made are known, comparing ESTs identified in libraries made from different tissues allows identification of tissue specific gene expression patterns. Sequence information can be found electronically by gene, tissue, function or disease. The National Cancer Institute Cancer Genome Anatomy Project (NCI CGAP (Strausberg 2001)) database lists 5,394 unique human genes related with the keyword vascular and 145 associated with the process angiogenesis.

## Figures and Tables

**Table 1:** Korean plants with antiangiogenic potential.

Reference number	Plant species (and family)	Traditional uses	Part used	Previously reported activity	Reported chemical constituents
PB3699.1	*Acer mono* Maxim.	Leaves are an irritant and bark is astringent [28]	Leaves	The sap increases calcium ion absorption in mouse [29]	5-*O*-methyl*-(E)-*resveratrol 3-*O*-**β**-D-glucopyranoside; 5-O-methyl*-(E)-*resveratrol 3-*O-*β*-*D*-*apiofuranosyl-(1→6)-**β*-*D*-*glucopyranoside; quercetin, quercitrin; eriodictyol; naringenin; eriodictyol-7-*O-*α*-D-*glucopyranoside; 5,7-dihydroxychromone 7-*O-*α*-*D*-*glucopyranoside; naringenin 7-*O-*α*-*D*-*glucopyranoside [30]

PB4737A.1	*Adenophora racemosa *J. Lee and S. Lee (Campanulaceae)	—	Whole plant	—	—

PB4338.1	*Caryopteris incana *(Thunb.) Miq. (Verbenaceae)	In China for the relief of colds, coughs, and rheumatic pains [36]	Leaves and stems	Antioxidant and cytotoxic activity reported for plant constituents [37, 38]	Incanone; suyiol [37]; incanoside; verbascoside, isoverbascoside, phlinoside A, 6-*O*-caffeoyl-beta-D-glucose; incanoside C, incanoside D and incanoside E; **β**-D-fructofuranosyl-**α**-D-(6-*O*-[*E*]-sinapoyl) glucopyranoside [38, 39]; 8-*O*-acetylharpagide; 6′-*O-p*-coumaroyl-8-*O*-acetylharpagide; (3*R*)-oct-1-en-3-ol *O-*α**-*L*-arabinopyranosyl-(1′′→ 6′)-*O-*β*-*D*-*glucopyranoside; apigenin 7-O-neohesperidinoside; 6′-*O*-caffeoylarbutin; leucosceptoside A; phlinoside A; 6′-*O*-Caffeoyl-8-*O*-acetylharpagide; (3*R*)-Oct-1-en-3-ol *O-*β**-D-glucopyranosyl-(1′′→2′)-*O-*β* -*D-glucopyranoside; (3*R*)-Oct-1-en-3-ol *O-*α**-L-arabinopyranosyl- (1′′′→ 6′′)-*O-*β**-D-glucopyranosyl-(1′′→2′)-*O-*β** -D-glucopyranoside [40]

PB2906.2	*Cinnamomum japonicum *Sieb. (Theaceae)	—	Stems	—	—

PB3828.1	*Eurya japonica *Thunb. (Theaceae)	As an ornamental [41]	Leaves	—	cyanidin 3-glucoside; Cyanidin 3-O-(6′′-O-(4′′′-*O*-acetyl-*α*-L-rhamnopyranosyl)-*B*-D-glucopyranoslde) [42]

PB2552.1	*Reynoutria sachaliensis *(F. Schmidt) Nakai.(Polygonaceae)	Crops protection against phytopathogenic fungi [43]	Fruits	Antioxidant activity [44]	Emodin; emodin-8-*O-*β**-D-glucopyranoside; physcion-8-O-beta-D-glucopyranoside; quercetin-3-O-alpha-L-arabinofuranoside; quercetin-3-O-beta-D-galactopyranoside; quercetin-3-O-beta-D-glucuronopyranoside; anthraquinones, stilbenes [44]

PB2892.1	*Schisandra chinensis *(Turcz.) Bail. (Magnoliaceae)	Protective effect against deficits of the lung, liver, and gall bladder, alleviate cough and satisfy thirst [45]	Stems	Antihepatotoxic [46], enhance hepatic glutathione regeneration capacity [47], anti-inflammatory [45]	Lignans (schizandrin; gamma-schizandrin; gomisins A, B, C, D, E and F); nortriterpenoids (pre-schsanartanin and schindilactones A–C; schintrilactones A and B; wuweizidilactones A–F) [46]

(—): not reported. The complete list of the tested plants is available in supporting information.

**Table 2:** Expression of angiogenesis-regulating genes correlating with IC_50_ values of selected phytochemicals in the NCI panel of tumor cell lines (*R* > 0.3; *R* < −0.3). The full names of the abbreviations given are listed in Supplementary Table  1.

Compound	Direct correlation with gene expression (*R* > 0.3)	Inverse correlation with gene expression (*R* < −0.3)
Verbascoside	*MPDZ, MMRN, F3, PECAM1, DDAH2, *and *NOTCH3, DVL3 *	NOS2A, C1QR1, PML, and STC1
Emodin	*PECAM1, NID2 *	TIMP3, SNX17, TFPI2, SPR, and ANG
Apigenin	*NRCAM *	FGR2
Eriodictyol tetraacetate	*FGFR2, SST, TEK, PML, *and *ANGPTL3 *	EFEMP1, C1QR1, PECAM1, STC1, and TGFB1
Resveratrol	*TIMP3, EphA2, PLT, PIN, *and *COL4A2 *	FN1, PECAM1, PML, ABCG1, and CXCR4
Axitinib (control drug)	*COL5A1, BPR2 *	THBS4

**Table 3:** Separation of clusters of the NCI cancer cell lines obtained by hierarchical cluster analysis for selected phytochemicals from antiangiogenic plants derived from traditional Korean medicine. The log_10_ IC_50_ median values (M) of each compound were used as cutoff values to define cell lines as being sensitive or resistant. *P* > 0.05 was considered as not significant (*χ* ^2^ test).

Compounds	Clusters	Sensitive	Resistant	*P*-value (*χ* ^2^ test)
Verbascoside	Partition*	≤−4.278	>−4.278	*P* = 3.35497 × 10^−4^
	Cluster 1	3	14	
	Cluster 2	4	9	
	Cluster 3	9	1	
	Cluster 4	8	1	

Emodin	Partition*	≤−4.607	>−4.607	P = 0.0142
	Cluster 1	7	2	
	Cluster 2	5	1	
	Cluster 3	8	15	
	Cluster 4	8	4	
	Cluster 5	1	6	

Apigenin	Partition*	≤−4.543	>−4.543	P = 0.02628
	Cluster 1	2	9	
	Cluster 2	5	4	
	Cluster 3	9	1	
	Cluster 4	6	7	

Eriodictyol tetraacetate	Partition	≤−4.358	>−4.358	n.s.**
	Cluster 1, 2, 3	6	2	
	Cluster 4	2	5	

Resveratrol	Partition*	≤−4.223	>−4.223	n.s.**
	Cluster 1	3	2	
	Cluster 2	9	5	
	Cluster 3	5	4	
	Cluster 4	3	10	
	Cluster 5	4	3	

Axitinib (control drug)	Partition*	<−5.015	>−5.015	n.s.**
	Cluster 1	10	15	
	Cluster 2	4	6	
	Cluster 3	15	8	

*log_10_ IC_50_ (M).

**n.s.: not significant (*P* > 0.05).

**Table 4:** *In silico* molecular docking to VEGFR1 and VEGFR2 of selected phytochemicals from antiangiogenic plants derived from traditional Korean medicine. (Residues marked bold are the drug binding residues).

Receptors	Compounds	Lowest energy of docking (kcal/mol)	Mean binding energy (kcal/mol)	Residues involved hydrogen bond interaction with the ligand	Number of residues involved in hydrophobic interaction with ligand
VEGFR1	Axitinib (control drug)	−12.71	−12.38	**Glu 878, Cys 912**, Glu 910, **Asp 1040**	12
	Eriodictyol tetraacetate	−9.92	−9.27	**Asp 1040**	14
	Quercetin	−9.01	−8.51	**Glu 878**, Glu 910, **Cys 912**	9
	Apigenin	−8.85	−8.56	**Glu 878,Cys 912, Asp 1040**	11
	Resveratrol	−7.89	−7.72	Lys 861, **Glu 878,Cys 912**	10
	Emodin	−7.30	−7.30	Leu 833, Glu 910, **Cys 912**	10
	Verbascoside	−6.93	−4.95	Arg 1021, Asp 1022, **Asp 1040**	11

VEGFR2	Axitinib (control drug)	−12.39	−12.20	Glu 917, **Asp 1046**	14
	Eriodictyol tetraacetate	−10.56	−9.85	Ala 1050	18
	Verbascoside	−9.99	−5.33	His 816, Thr 916, **Asp 1046**, Ala 1050	19
	Apigenin	−9.04	−9.01	**Lys 868, Cys 919, Asp 1046**	11
	Quercetin	−8.29	−8.18	Ala 881, Glu 885, Ile 1025, Ile 1044	11
	Resveratrol	−8.15	−8.05	**Lys 868, Cys 919, Asp 1046**	9
	Emodin	−7.63	−7.35	Ile 1025, **Asp 1046**	9
