# Supplementary material for: Antiangiogenic Activity and Pharmacogenomics of Medicinal Plants from Traditional Korean Medicine
Source: Evid Based Complement Alternat Med. 2013 Jul 22;2013:131306. doi: 10.1155/2013/131306 (PMC3736538; doi:10.1155/2013/131306)
Supplement: Supplementary file 1 — EST sequencing and electronic databases are used for gene profiling by F. V. Peale Jr. (Peale and Gerritsen 2001). Briefly, EST sequencing is creating cDNA libraries from tissues or cells of interest, randomly select clones, and perform a single sequencing reaction on each clone (Adams et al. 1991). The EST approach has been used to identify what can be the most expressed sequences in the human genome. This information can be applied in many ways, for instance, the tissues from the EST libraries are made are known, comparing ESTs identified in libraries made from different tissues allows identification of tissue specific gene expression patterns. Sequence information can be found electronically by gene, tissue, function or disease. The National Cancer Institute Cancer Genome Anatomy Project (NCI CGAP (Strausberg 2001)) database lists 5,394 unique human genes related with the keyword vascular and 145 associated with the process angiogenesis. [file 131306.f1.pdf]

Anti-angiogenic activity and pharmacogenomics of medicinal plants from traditional Korean medicine

Ean-Jeong Seo*, Victor Kuete*, Onat Kadioglu, Benjamin Krusche, Mira Oswald, Sven Schröder, Henry Johannes Greten, Joachim Arend, Ik-Soo Lee, Thomas Efferth

- -

Supplementary Table: Genes involved in angiogenesis (taken from: Peale Jr FV, Gerritsen ME. Gene profiling techniques and their application in angiogenesis and vascular development. J Pathol 2001; 195: 7–19.

Gene Symbol	Pattern ID	Genbank Accession	Gene Name
TIMP3	GC19231	AA043969	Tissue inhibitor of metalloproteinase 3 (Sorsby fundus dystrophy, pseudoinflammatory)
THBS4	GC15218	N80868	Thrombospondin 4
HGF	GC14697	N62083	Hepatocyte growth factor (hepapoietin A; scatter factor)
HGF	GC17092	AA037786	Hepatocyte growth factor (hepapoietin A; scatter factor)
THBS1	GC10044	AA056585	Thrombospondin 1
ACTG2	GC9740	AA053297	actin, γ2, smooth muscle, enteric
AXL	GC11900	T91043	AXL receptor tyrosine kinase
FGFR2	GC10638	R38513	Fibroblast growth factor receptor 2
FGFR2	GC17772	W87790	Fibroblast growth factor receptor 2
TIMP2	GC15806	W49722	Tissue inhibitor of metalloproteinase 2
EphA2	GC17968	AA001368	Ephrin A2
SST	GC16150	W72209	Somatostatin
TEK	GC12925	H44411	TEK tyrosine kinase, endothelial (venous malformations, multiple cutaneous and mucosal)
BMB6	GC12254	R27799	Bone morphogenetic protein 6
TGFB2	GC14551	N48082	Transforming growth factor, β2
NOS2A	GC13640	H67843	Nitric oxide synthase 2A
VEGF	GC15550	W19225	Vascular endothelial growth factor
VEGF	GC9897	AA045836	Vascular endothelial growth factor
NOS3	GC12213	R21248	Nitric oxide synthase 3
FGFR1	GC10555	T66144	Fibroblast growth factor receptor (fms-related tyrosine kinase 2, Pfeiffer syndrome)
FGFR1	GC12641	R54846	Fibroblast growth factor receptor (fms-related tyrosine kinase 2, Pfeiffer syndrome)
CD44	GC11989	T91563	CD44 antigen (homing function and Indian blood group system)
SSP1	GC13722	H93048	Secreted phosphoprotein 1 (osteopontin, bone sialoprotein, early T-lymphocyte activation 1)
SSP1	GC16786	AA021512	Secreted phosphoprotein 1 (osteopontin, bone sialoprotein, early T-lymphocyte activation 1)
SELE	GC12936	H39991	Selectin E (endothelial adhesion molecule 1)
FN1	GC12293	R36451	Fibronectin 1
FN1	GC12570	H03907	Fibronectin 1
FN1	GC15675	W15432	Fibronectin 1
FN1	GC10483	AA057805	Fibronectin 1
MMP9	GC11583	T41204	Matrix metalloproteinase 9 (gelatinase B, 92kDa type IV collagenase)
COL1A2	GC10023	AA056542	Collagen, typeI, α2
COL3A1	GC9808	AA044829	collagen, type III, α1 (Ehlers-Danlos syndrome type IV, autosomal dominant)
EFEMP1	GC18383	AA030000	EGF-containing fibulin-like extracellular matrix protein 1
EFEMP1	GC18944	AA040443	EGF-containing fibulin-like extracellular matrix protein 1
MPDZ	GC14622	N71935	Multiple PDZ domain protein
ARG2	GC13080	R94927	Arginase, type II
TRB	GC15271	N79985	T-cell receptor β locus
PLAT	GC17776	W89129	Plasminogen activator, tissue

ADM	GC16644	AA017304	Adrenomedullin
PIN	GC18907	AA040248	Dynein, cytoplasmic, light polypeptide
C1QR1	GC19150	AA044236	Complement component 1, q subcomponent, receptor 1
IL8	GC15899	W45324	Interleukin 8
COL1A1	GC17894	W90360	Collagen, type I, α1
MMRN	GC16095	W73053	Multimerin
F3	GC12286	R34833	Coagulation factor III (thromboplastin, tissue factor)
SPARCL1	GC18568	AA033762	SPARC.like 1 (mast9, hevin)
CTGF	GC19343	AA044993	Connective tissue growth factor
COL4A2	GC10048	AA056692	Collagen, type IV, α2
MGP	GC16120	W72057	Matrix Gly protein
CHD5	GC13933	N22448	Cadherin 5, type 2, VE-cadherin (vascular epithelium)
NRCAM	GC10822	R45583	Neuronal cell adhesion molecule
NRCAM	GC14758	N59824	Neuronal cell adhesion molecule
PLAU	GC19033	AA040727	Plasminogen activator, urokinase
TFPI2	GC16650	AA017446	Tissue factor pathway inhibitor 2
TFPI2	GC18167	AA011474	Tissue factor pathway inhibitor 2
PECAM1	GC11837	T72992	Platelet/endothelial cell adhesion molecule (CD31 antigen)
PECAM1	GC10029	AA056412	Platelet/endothelial cell adhesion molecule (CD31 antigen)
ANGPTL2	GC16029	W73148	Angiopoietin-like 2
IL6ST	GC13860	H85457	Interleukin 6 signal transducer (gp130, oncostatin M receptor)
HPN	GC13241	GC13241	Hepsin (transmembrane protease, seine 1)
COL18A1	GC16530	AA009987	Collagen, type XVIII, α1
SNX17	GC10384	AA053676	Sorting nexin 17
MDK	GC16107	W72586	Midkine (neurite growth-pro,moting factor 2)
SERPINE1	GC19310	AA046659	Serine (or cys) proteinase inhibitor, clase E (nexin, plasminogen activator inhibitor type 1), 1
MMP11	GC18601	AA034203	Matrix metalloproteinase 1 (stromyelysin 3)
MMP11	GC15801	W49497	Matrix metalloproteinase 1 (interstitial collagenase)
FST	GC12080	R01927	Follistatin
DDAH2	GC15208	N81116	Dimethylarginine dimethylaminohydolase 2
CD34	GC13318	H72113	CD34 antigen
NOTCH3	GC18151	AA007598	Notch homologue 3 (*Drosophila*)
CYR61	GC19161	AA044574	Cystene-rich, angiogenic inducer, 61
PML	GC12650	R55296	Promyelocytic leukemia
PML	GC15315	N93133	Promyelocytic leukemia
PML	GC17137	AA039542	Promyelocytic leukemia
MMP2	GC12462	R79223	Matrix metalloproteinase 2 (gelatinase A, 72kDa type IV collagenase)
NOTCH4	GC16035	W74767	Notch homologue 4 (*Drosophila*)
C6orf9	GC11019	R59617	Chromosome 6 open reading frame 9
BST2	GC12646	R54976	Bone marrow stromal cell antigen 2
ITGA5	GC16514	W92322	Integrin, α5 (fibronectin receptor, α polypeptide)
THY1	GC12929	H28104	Thy-1 cell surface antigen
THY1	GC18445	AA031264	Thy-1 cell surface antigen
BMP1	GC12413	R71212	Bone morphognetic protein 1
BMP1	GC17017	AA029677	Bone morphognetic protein 1
M11S1	GC16436	W94794	Membrane compenent, chromosome 11, surface marker 1
COL5A1	GC18776	AA037002	Collagen, type V, α1
BMPR2	GC13867	H96519	Bone morphognetic protein receptor, type II (serine/threonine kinase)
INHBA	GC14152	N27159	inhibin, βA (activin AB a polypeptide)
GJA4	GC15957	W69079	Gap junction protein, α4, 37kDa (connecin 37)
DVL3	GC16240	W79440	Dishevelled, dsh homologue 3 (*Drosophila*)
DVL3	GC19142	AA044175	Dishevelled, dsh homologue 3 (*Drosophila*)
STC1	GC12616	R48580	Stanniocalcin 1
ABCG1	GC13298	H68928	ATP-binding cassette, subfamily G (WHITE), member 1
TGFB1	GC12433	R76212	Transforming growth factor β1
FGF2	GC15733	W44678	Fibroblast growth factor 2 (basic)
FGF2	GC15826	W52295	Fibroblast growth factor 2 (basic)
FGFR2	GC10638	R38513	Fibroblast growth factor receptor 2
FGFR2	GC17772	W87790	Fibroblast growth factor receptor 2
EDG1	GC15407	N93476	Endothelial differentiation, spingolipid G-protein-coupled receptor 1
CXCR4	GC12441	R78620	Chemokine (C-X-C motif), receptor 4 (fusin)
NID2	GC16008	W70102	Nidogen 2
PGF	GC18503	AA031377	Placental growth factor, vascular endothelial growth factor-related protein

MCAM	GC12722	H28333	Melanoma cell adhesion molecule
MCAM	GC19206	AA042901	Melanoma cell adhesion molecule
SPR	GC16989	AA026314	Sepiapterin reductase (7,8-dihydrobiopterin:NADP$^+$ oxidoreductase
VEGFB	GC18978	AA040199	Vascular endothelial growth factor B
VEGFC	GC15494	N94399	Vascular endothelial growth factor C
FLT1	GC14477	N47214	Fms-related tyrosine kinase 1 (vascular endothelial growth factor/vascular permeability factor receptor
ANGPTL3	GC11804	T73442	Angiopoietin-like 3
HIF1A	GC15805	W47003	Hypoxia-inducible factor 1, α subunit (basic helix-loop-helix transcription factor)
ANG	GC18183	AA011614	Angiogenin, ribonuclease, RNase A family, 5
